# Toward an Automatic Quality Assessment of Voice-Based Telemedicine Consultations: A Deep Learning Approach

**DOI:** 10.3390/s21093279

**Published:** 2021-05-10

**Authors:** Maria Habib, Mohammad Faris, Raneem Qaddoura, Manal Alomari, Alaa Alomari, Hossam Faris

**Affiliations:** 1 Altibbi, King Hussein Business Park, Amman 11831, Jordan; maria.habib@altibbi.com (M.H.); mohammad.faris@altibbi.com (M.F.); manal@altibbi.com (M.A.); alaa.alomari@altibbi.com (A.A.); 2 Faculty of Information Technology, Philadelphia University, Amman 19392, Jordan; rqaddoura@philadelphia.edu.jo; 3 King Abdullah II School for Information Technology, The University of Jordan, Amman 11942, Jordan; 4 School of Computing and Informatics, Al Hussein Technical University, Amman 11831, Jordan

**Keywords:** Altibbi, deep learning, feature extraction, telehealth, telemedicine, voice-based signals, signal processing

## Abstract

Maintaining a high quality of conversation between doctors and patients is essential in telehealth services, where efficient and competent communication is important to promote patient health. Assessing the quality of medical conversations is often handled based on a human auditory-perceptual evaluation. Typically, trained experts are needed for such tasks, as they follow systematic evaluation criteria. However, the daily rapid increase of consultations makes the evaluation process inefficient and impractical. This paper investigates the automation of the quality assessment process of patient–doctor voice-based conversations in a telehealth service using a deep-learning-based classification model. For this, the data consist of audio recordings obtained from Altibbi. Altibbi is a digital health platform that provides telemedicine and telehealth services in the Middle East and North Africa (MENA). The objective is to assist Altibbi’s operations team in the evaluation of the provided consultations in an automated manner. The proposed model is developed using three sets of features: features extracted from the signal level, the transcript level, and the signal and transcript levels. At the signal level, various statistical and spectral information is calculated to characterize the spectral envelope of the speech recordings. At the transcript level, a pre-trained embedding model is utilized to encompass the semantic and contextual features of the textual information. Additionally, the hybrid of the signal and transcript levels is explored and analyzed. The designed classification model relies on stacked layers of deep neural networks and convolutional neural networks. Evaluation results show that the model achieved a higher level of precision when compared with the manual evaluation approach followed by Altibbi’s operations team.

## 1. Introduction

Providing a high quality of service in telehealth is a leading cause of success and a prime objective for clinicians and providers of telemedicine. Generally, the quality of telemedicine services can be influenced by various factors related to the patients, the physicians, and the environment. This includes but is not limited to patient cooperation, demographic and health situations, physician satisfaction, and the healthcare system and resources. Maintaining a high quality of telemedicine services is a subjective process, and differs among facilities. Some consider it from the perspective of covering the patient’s needs efficiently and effectively in a way that meets the provider’s satisfaction. Others have identified that the quality of the service is fulfilled by providing the right service at the right time, in the right place, for the right patient, for the right price. Further, others believe that maintaining a high quality of service can be done by delivering the care to a degree that exceeds the patients’ expectations [1]. Roughly speaking, to ensure a high quality of telemedicine services, such systems should preserve the availability, accessibility, timeliness, privacy, and the confidentiality of service and caring, and should provide responsive communication, accuracy, reliability, as well as the improvement of patient quality of life [2]. Quantifying the quality of medical services in the case of recorded consultations is not easy. In such situations, the recordings contain the voices of the doctor and the patient, where they might be speaking in different dialects of the language. Meanwhile, capturing their attitudes, feelings, or reactions based on the recorded audio is challenging. As the recorded voice is an acoustic signal rich in spectral features and other linguistic and phonetic structures, Roy et al. intended to assess the quality of medical consultations to convey the speakers’ attitude [3].

At present, with the epidemiological circumstance of the coronavirus disease (COVID-19) that the whole world is experiencing, the world is witnessing a rapid increase in telehealth and telemedicine services. Manually evaluating the quality of a large number of provided consultations on a daily basis is cumbersome and inefficient. Alternatively, automating the process of quality evaluation of such services is a substantial challenge that is sought to reduce efforts and improve the service. Recently, artificial intelligence techniques, including machine and deep learning methods have been an integral part of different systems in diverse domains, which intelligently mimic human abilities. The automation of the quality evaluation process of medical consultations is an example of the utilization of artificial intelligence in telehealth.

Telehealth platforms have flourished during the COVID-19 pandemic. A well-known platform in the MENA region is Altibbi (https://altibbi.com, accessed on 5 May 2021). It is a digital health platform that provides telemedicine and telehealth services for individuals in the MENA region, where the main language is Arabic. The primary objective of Altibbi is to make high-quality telemedicine consultations attainable and available to all in the MENA region. As delivering high-quality care is the core interest of Altibbi, Altibbi’s Expert Doctors are assigned to provide a structured and constructive evaluation of the conducted consultations regularly by checking several aspects, such as the doctor’s attitude, approach skills, how they ask about the patient’s medical history and symptoms, and their general patient management skills. This process of evaluation is performed manually, where the Expert Doctors have to listen to a large number of consultations to find the ones of low quality. For example, they need to listen to 100 consultations to identify around 20 low-quality consultations. However, since the outbreak of the pandemic, Altibbi started to witness a dramatic increase in the number of consultations daily (e.g., Altibbi received around 10,000 consultations per day in Q1-2021). Therefore, automating the process of quality evaluation will ease the process of finding low-quality consultations without requiring the operations team to listen to a very large number of consultations.

Motivated by this problem, this paper proposes an approach to automate the process of consultation evaluation based on deep learning. The recorded consultations in the Arabic language are stored in audio format. The proposed model analyzes the consultations from two perspectives: the speech signals, and the transcripts of the consultations. The signal-based analysis is concerned with extracting various features to encode the waveforms of the audio in more compact representations. The extraction of features from speech signals can be performed in the time domain, frequency domain, or time-frequency domain, or they can be other higher-level features such as homogeneity and timbre. The time-domain features (the temporal features) extracted from the waveforms usually encompass the characteristics of amplitude, power, and rhythm features. The frequency-domain features (spectral features) extracted from the spectrograms reveal information about the chroma, tonality, brightness, the Fourier transform, and the spectrum shape. Usually, the process of extracting features is done in several steps, where it is first required to segment the signal into chunks with overlapping windows, followed by a series of mathematical transformations and operations to extract the features of interest [4]. In transcript-based analysis, text-based features are extracted by using neural embedding models. However, this is subject to different obstacles as the recordings are in Arabic and of different dialects, where transcribing them to extract text-based features is a challenging process.

Regarding the signal-based analysis, three spectral features are extracted, which include the Mel-frequency cepstral coefficients (MFCCs), the Mel spectrogram, and the zero-crossing rate. The objective of the MFCCs and Mel spectrogram is to produce compact representations of acoustic characteristics of speech which encompass the frequency and power spectrums. Meanwhile, the zero-crossing rate quantifies the silent (unvoiced) periods in the conversations. Spectral features are used in different domains to characterize and sense acoustic signals. For example, the Fourier transform spectrum was used to recognize faults in motors by analyzing their acoustic signals in [5]. Meanwhile, the time and wavelet features of signals generated from monitoring sensors were used to identify the breakage of tools and improve the hole quality in micro-drilling in [6].

On the other hand, conversations are assessed textually by converting the speech into text and then analyzing it using pre-trained word embeddings and deep convolution neural network models. The conversion of speech into text is done using Amazon Transcribe, which is an automatic speech recognition service. The generated transcripts are vectorized and modeled using a pre-trained word embedding model for the Arabic language, “AraVec”, where the objective is to represent the transcripts in a suitable format for the input of the convolution network. The extracted features are used to formulate a binary classification problem for the quality prediction of consultations. The extracted features are utilized in three approaches: one is to analyze the signal-based features alone, the second is to process the features extracted from the transcripts, and the third is to combine both as sub-models and combine their output. Two variants of the neural network are utilized, a deep-stacked neural network and a deep-stacked convolutional network. The convolutional neural network is a type of artificial neural network but uses convolution and pooling layers to create feature maps. The structure of both models is described in detail in the methodology section. All models are evaluated using the precision, recall, F1-score, accuracy, and loss, which show encouraging results in the identification of low-quality consultations compared to the manual approach followed by the operations team.

The key contributions of this paper are as follows.

- We develop a model to automate the quality prediction of medical consultations. Particularly, the contribution at this point is at the feature engineering and model development levels. The model combines spectral features from the signals and text-based features from the transcripts, which will then be used to train different structures of deep and convolutional learning models.
- We reinforce the advantages of artificial intelligence in telemedicine. The development of an automatic quality assessment model reduces the effort and time for evaluating the consultations manually by the operations team. Besides, in pandemic situations such as the emergent COVID-19 pandemic, such an approach can enhance the quality of the service and better serve callers (patients).

The rest of the paper is organized as follows. Section 2 presents the related works in the literature. Section 3 presents the problem description and the motivation for the work. Section 4 introduces the theories of the used algorithms and concepts. Section 5 illustrates the methodology, including the dataset, the signal-based approach, the transcript-based approach, the experimental settings, and the evaluation criteria. Section 6 shows and discusses the obtained results. Section 7 provides the conclusions and future perspectives.

## 2. Related Works

Over the years, different studies attempted to automate the process of evaluating the quality of recorded calls across various domains, such as in telecommunication via call centers [7]. The quality of phone/recorded calls can be determined from different cornerstones, including the attitudes of speakers, their cooperation, the correctness of delivered information, voice tone and sentiment, oral proficiency, communications, and listening skills. Several studies evaluated the speech status based on the sentiments. For instance, Popovic et al. [8] proposed an automatic speech and sentiment detection approach for multi-party conversations. de Pinto et al. [9] created an emotion understanding model for recorded speech using deep neural networks and MFCCs vectors. A multimodal approach for text and audio was developed by Yang et al. [10] to extract the sentiments. Even though these studies depended only on the sentiment, other aspects should be considered to formulate the overall quality.

One of the earlier studies concerned with the automatic quality evaluation of calls without human interactions was conducted by Bae et al. [11]. They proposed a web-based tool to analyze the voices of customers at a call center to determine when there was a complaint. Their method relied on statistical and data mining methods to capture patterns encompassed in voices. In a different context, Takeuchi et al. [12] analyzed calls recorded from a rental car reservation office to inspect whether a caller would book a car or not by utilizing a trigger segment detection and expression extraction model. Garnier-Rizet et al. [13] developed a call-based evaluation tool “CallSurf” to enhance the efficiency of quality assurance systems in call centers by scoring recorded conversational speeches. The tool automatically transcribes the recordings and then extracts knowledge to assess their quality. Besides, Pandharipande and Kopparapu [14] created a new method to identify problematic conversations in call centers by using the speaking rate feature and modeling conversations as directed graphs to extract structural features.

In the banking domain, Pallotta et al. [15] utilized an interaction mining approach to enhance a call center analytic by employing a set of manually transcribed calls. Moreover, Kopparapu [16] proposed an automatic model for the identification of problematic calls in call centers. The authors extracted linguistic features from transcribed calls to recognize abnormal calls. Meanwhile, they referred to the limitations of the transcription process, as it is not accurate and incapable of identifying emotions accompanying phrases (i.e., if a phrase is spoken with gratitude or sarcasm). A distributed call monitoring framework was created by Karakus and Aydin [17] to evaluate the recorded calls of a customer service representative. The Hadoop MapReduce framework was used for the analysis, with the calls dataset transcribed using the Google Speech API. A list of slang words was integrated into the monitoring model with text similarity methods to label low-quality calls. An automated speech scoring system was developed by Chen et al. [18] to assess the readiness of English test takers to join a school where English is the primary language. Different variants of recurrent and convolutional neural networks were utilized to extract linguistic and acoustic features (including pitch, intensity, and word duration). Meanwhile, a linear regression method was used at the final stage to generate the scores. Furthermore, Perera et al. [19] created automatic evaluation software for assessing the quality of agents’ voices in a contact center. The aim was to minimize the bias of human evaluators by developing a model that employed the speech rate, the intensity of the voice, and the emotional state of the voice. Several non-linguistic features were extracted, such as pitch, zero-crossing rate, MFCC, and others. Meanwhile, the support vector machine algorithm was used as the quality-based classifier. A multimodal approach was proposed by Ahmed et al. [20] to promote the evaluation process of a call center agent. Statistical and spectral features were extracted from speech using the OpenSmile library, while other text-based features were extracted from transcripts using a hybrid convolution and recurrent neural network. Enhancing the transcription criteria and the text-based language modeling are two areas for improvement to extend their research.

To the best of our knowledge, no previous works studied the automatic quality assessment of recorded speech/calls in the Arabic language in the telemedicine context. The challenges associated with processing Arabic transcripts and the lack of medical call (audio-based) datasets make the problem critical and difficult to implement. This paper attempts to fill the gap by proposing an automatic quality prediction model for evaluating the quality of Altibbi’s medical consultations, where the data are collected from Altibbi’s databases and labeled manually by Altibbi’s expert doctors.

## 3. Problem Definition

Altibbi’s Call Doctor service is a primary care telemedicine service available for users in the MENA region for a low charge. This service aims to address the diagnosis, treatment, and monitoring of patients by providing patients with immediate and private access to primary care physicians within the region through GSM conferencing or synchronous chats.

The service aims to provide care as safely and effectively as traditional in-person visits. Whereas telemedicine might offer convenience and a lower cost alternative from the patients’ perspective, clinicians are required to deal with a new care delivery module that limits the amount of information received from patients. As part of Altibbi’s quality assurance process, consultations are sampled and evaluated based on different criteria, including the quality of the medical information provided to the user, the doctor’s attitude and behavior, and if there is a breach of any of Altibbi’s policies which violates the doctor’s agreement or ethics.

A primary role of “Altibbi Expert Doctors” is to scrutinize the quality of the medical information presented to the user. Altibbi works with Expert Doctors from different specialties to monitor, evaluate, and provide regular feedback to Altibbi doctors to improve the quality of care. All Altibbi doctors are closely monitored for two weeks after they join Altibbi. All their consultations are viewed and evaluated by experts against defined measures. If no errors are detected, they are allowed to take a higher number of medical consultations, and then they are evaluated regularly thereafter. Accordingly, a random sample is taken for each doctor and sent to the Expert Doctors, in which the experts do a structured evaluation and send constructive feedback to the doctor. Expert Doctors evaluate the consultations based on different bases, such as the doctor’s attitude and skills in problem-solving, taking a comprehensive relative medical history, asking about symptoms, patient management, reaching a differential diagnosis, and referring the patient if needed.

Therefore, the Expert Doctors assign a level-of-quality for each evaluated consultation. These levels were proposed and discussed internally with the doctors by the operations director to capture the doctors’ overall impression of the quality of the consultation, and to indicate the severity or harmfulness of the detected mistakes or errors. The levels-of-quality utilized in the evaluation criteria are as follows. “Excellent” indicates nothing is missing, or that there are minor points missing which do not influence the final diagnostic decision. An “Excellent” score is represented by the numerical value “5”. The second level is “Good” (“4”), where essential points are missing but no harm is done to the patient. Third is “Acceptable consultation”, labeled by the value“3”. Fourth is “Poor consultation”, where there incomplete history is obtained and/or weak management is conducted; this is assigned a numerical value of “2”. Finally, the lowest level is “Very poor consultation” (“1”), where the doctor engages in wrong and harmful management or prescribes an incorrect drug.

This evaluation is made in order to aid the medical operations team in recognizing consultations that need escalation. However, randomly sampling consultations to pinpoint which of them are of low quality is not an efficient method, and requires the operations team to listen to a large number of consultations to find the low-quality consultations. For example, if the percentage of low-quality consultations is 20%, in a random sample the operations team has to check 100 consultations to find 20 low-quality items in the best case scenario. However, the operations team needs a smart model to find those 20 consultations. Therefore, a more systematic and automatic procedure is required to handle this problem, which is the objective of this paper—to build a deep-learning-based model to assess the quality of consultations and help the operations team in finding the low-quality consultations.

## 4. Background

This section gives a brief description of the theories and algorithms needed to implement the automatic quality approach as presented in the remaining sections. It demonstrates the extracted spectral features (including the spectrogram, MFCCs, and the zero-crossing rate), the process of Amazon transcription for retrieving the consultation transcriptions, and the concepts behind the deep forward neural networks and the convolution neural networks.

### 4.1. MFCCs and Mel Spectrogram

MFCCs [21] are a representation of the enclosed envelope of the power spectrum that manifests the characteristics of the human voice and the vocal tract. These coefficients characterize the Mel-frequency cepstrum, which is known as the “spectrum-of-a-spectrum”, and is required to spot the periodic components of a signal in the time domain as peaks in a new domain referred to as the “quefrency” domain. The process of extracting the MFCCs consists of several mathematical transformations of the signal from the time domain to the frequency domain, to the quefrency domain. This comprises the Fourier transform and the discrete cosine transforms to obtain the log-magnitude representation of the spectrum [22,23]. The generation of MFCC vectors consists of the following steps:

- Pre-emphasizing the input signal to remove unwanted or high frequencies.
- Framing and windowing the signal, where the objective is to divide the signal into a sequence of short overlapping frames to ensure that they are stationary, where a stationary signal reflects the true statistical and temporal characteristics. The windowing is often performed using rectangular windows as the Hamming window that conceals the potential of distorted segments found at the boundaries of the windows by smoothing them.
- Applying the Fourier transform of the signals to convert them from the time domain to the frequency domain to represent them in terms of their statistical and spectral features.
- Applying filter banks (“Mel filters”) to generate frames in the Mel scale.
- Computing the logarithmic value of the magnitude of powers resulted from the Mel filters.
- Calculating the spectrum of the results produced from the previous step by applying the discrete cosine transform (DCT) that results in cepstral coefficients as represented by Equation (1), where *n*∈ {0, 1, *…* C-1}, c(n) represents the cepstral coefficients, and *C* is the number of MFCCs.
  (1) $$c\left(n\right)=\sum_{0}^{M-1}log_{10}\left(s\left(m\right)\right)cos\left(\frac{\pi n(m-0.5)}{M}\right)$$
  Conventionally, the MFCCs are from 8 to 13 features, however, those 13 coefficients exhibit static features of the respective frames apart. The generation of more temporal features is done by finding the first and second derivatives of the cepstral coefficients known as the delta and delta-delta features. Accordingly, the MFCCs are extended from 13 to 39 coefficients.

The MFCCs have been widely utilized to model the acoustic information of auditory signals over different applications, such as audio content classification and voice recognition [24,25,26].

**Figure 1:**
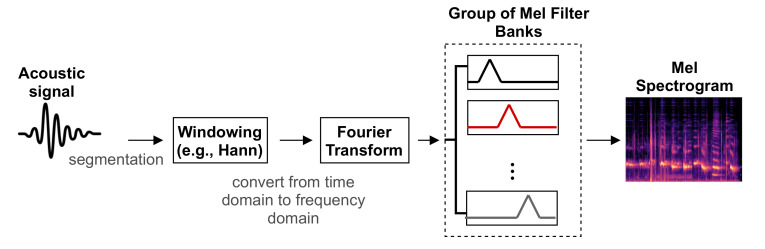
The process of extracting the Mel spectrogram from an acoustic signal, where the output of each Mel filter is summed then combined to create the Mel spectrogram, which is visualized in terms of the amplitude of the frequency components over time.

On the other hand, the spectrogram is a visual representation of signal strength in terms of the signal’s loudness or intensity, which shows the variation of the frequencies of acoustic signals over time. Often, the frequency is presented on the *y*-axis and the time on the *x*-axis, while the color of the plot reveals the intensity. The utilized spectrogram in this paper is the Mel spectrogram, where the frequency is mapped and presented in the Mel scale. Figure 1 displays the process of extracting the spectrogram features from auditory signals, in which the signals are divided into short overlapping windows, then converted into the frequency domain using the Fourier transform, then the Mel filter banks are used to produce the spectrogram envelope.

### 4.2. Deep Neural Networks (Convnet)

**Figure 2:**
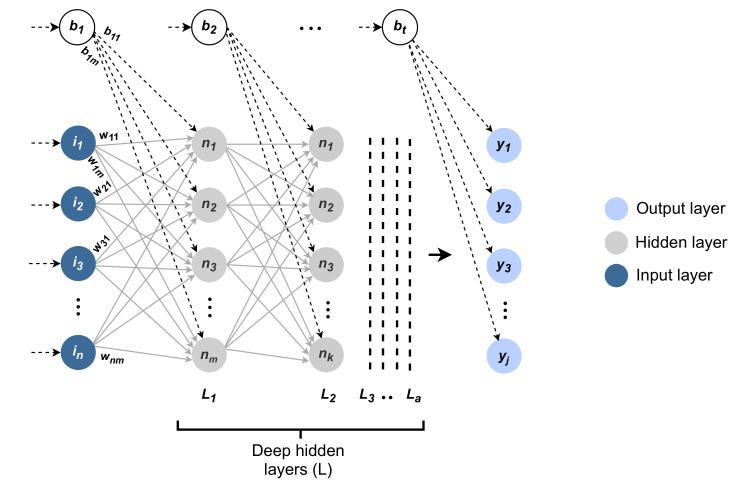
The anatomy of a deep neural network model, *a* is the number of hidden layers.

The deep neural network consists of several layers of neurons that are connected and structured in a shape that resembles the neural networks of the human brain. It is supposed to mimic how the information flows and is inferred across the brain, while the structure of the neural network is intended to decode the input data and extract potential hidden relationships from it iteratively by tuning the network parameters [27]. Figure 2 shows the anatomy of the deep neural network. The deep neural network includes the input layer, the hidden layers, and the output layer, with a set of weights and biases.

The input layer has the values of *n* features for each instance, which are represented by the set $I=i_{1},i_{2}\ldots i_{n}$. The output layer includes the predicted value for *j* possible labels, which are represented by the set $Y=y_{1},y_{2}\ldots y_{j}$. The hidden layers find the relationships between the input values *I* and the output values *Y* by computing the value for each hidden neuron at each hidden layer. A neuron has two major mathematical operations; the first is the summation, and the second is the activation. However, the hidden layers can have a different or equal number of hidden neurons. For instance, in Figure 2, *m* neurons can be observed for one hidden layer, and *k* neurons can be observed for another layer.

The basic form of the deep neural network calculates the values of the neurons for the first hidden layer by running an activation function of the sum of products between each input value *I* and the weight connecting the input value with the neuron while adding a bias value. Accordingly, the sum of products is given by Equation 2, where *n* is the number of features, *i* is the input, *w* is the weight, and *b* is the bias.

(2) $$z=\sum_{x=0}^{n}w_{x}i_{x}+b$$

The activation part of a neuron is a linear or non-linear activation function, which is adopted primarily to generate a non-linear output of the neuron and allows the network to back-propagate errors and optimizes the parameters. A popular activation function is the hyperbolic tangent function “tanh”.

Then, the output of each neuron of the first layer is considered as an input value to the next hidden layer, and so on, until the output layer is reached, which finds the predicted values of each label. The error values are observed by the difference between the predicted values and the actual values; meanwhile, the network adjusts the values of the weights using the back-propagation technique to produce a more accurate relationship between the input values and the output values.

Deep neural networks have been used by many applications and with different learning styles, including supervised and unsupervised approaches. These algorithms have robust capabilities when used to learn from large amounts of data, providing them with higher generalization power and the ability to handle and process complex datasets [28]. Neural network algorithms have been used in various applications, including intrusion detection [29,30], speech recognition [31,32], computer vision [33], autonomous cars [34,35], fraud detection [36,37], and healthcare [38], among others.

A convolutional neural network (CNN) is a type of deep neural network capable of analyzing data structured in a grid-like format (e.g., images). Meanwhile, CNNs are also adaptable with text and time-series data since they are one-dimensional grid structures. Roughly, a CNN is a neural network that applies the convolution operation in at least one of its layers. Typically, the topology of any CNN network employs convolution and pooling layers to generate feature maps. However, there is no one standard topology of CNN structure suitable for any problem. The architecture of a CNN (including the number of convolution layers, the size of the filters, and pooling layers) relies highly on the type of problem and the shape and nature of the data.

In general contexts, convolution is a mathematical operation that convolves two functions to generate another one; in the case of a CNN, this is a multiplication operation of two matrices to create another new matrix. Practically, the input type of a CNN is a multi-dimensional matrix (“tensor”), while the convolution operation is a discrete operation presented by the asterisk symbol *. It is important to note that the concept of the convolution operation is identical, and the computation does not differ, regardless of the size of the input dimension (i.e., one, two, or three dimensions). Equation (3) describes the mathematical definition of the convolution, where *x* is a two-dimension input matrix, *k* is a two-dimension kernel matrix, *n* and *m* are the width and height of the kernel, and f(t) is the convolution output that is called the feature map.

(3) $$f\left(t\right)=\left(x*k\right)\left(i,j\right)=\sum_{i}^{n}\sum_{j}^{m}x\left(n,m\right)k\left(i-n\right)\left(j-m\right)$$

In the context of one-dimensional input to the CNN (e.g., text), the length of the input is the number of words from all sentences fed in batches to the network. The width of the input matrix equals the embedding dimension of the words. Thus, the width of the convolution kernel is the same as the embedding dimension size, while its height is variable to encompass a different number of words when creating the feature maps. The primary objective of filters (i.e., convolution kernels) is to act as feature detectors, where each filter is responsible for extracting a different kind of feature. The resultant feature maps of the convolution pass through a non-linear activation function, such as the rectified linear unit (ReLU). The ReLU is a non-linear, non-saturating function that avoids the vanishing problem of gradients, and simply converts negative values to zero, as shown in Equation (4).

(4) ReLU(x)=max(0,x)

The final stage after the convolution and non-linear activation is the pooling, which is used as a subsampling operation to reduce the dimensionality while maintaining the most informative features. The pooling operation might be max-pooling, average-pooling, or sum-pooling. In max-pooling, the highest value of each produced feature map is preserved, where the output of the pooling operation is a fixed-length vector that equals the number of generated feature maps. The same is true for average and sum pooling, where the average value or the summation is calculated instead of the maximum.

**Figure 3:**
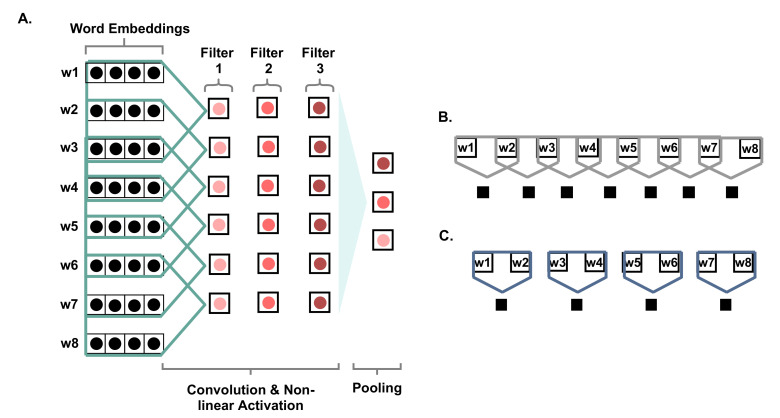
Description of the convolutional neural networks. In (**A**), the filter size is 3 and the stride is 1, (**B**) the filter size is 2 and the stride is 1, and (**C**) the filter size is 2 and the stride is 2.

Figure 3 shows the steps of applying the CNN for text. The figure presents an input sentence of eight words, where the dimension of the word embedding is roughly considered to be four. A convolution layer is applied to the input embeddings by applying three filters of size three and a stride value of one. According to the figure, they are presented by the vectors underneath the three filters, where they are one-dimensional with size 6. The pooling operation is the maximum value from each feature map, given that the pooling type is max-pooling. Figure 3A shows the convolution of three filters of size three and moving one unit of stride at a time, covering three words each time. In contrast, Figure 3B shows a filter size of two and a stride of one, while Figure 3C presents a filter of size two and a stride of two. It can be noticed that a different number of feature maps can be generated for Figure 3A–C with sizes of six, seven, and four, respectively.

## 5. Methodology

**Figure 4:**
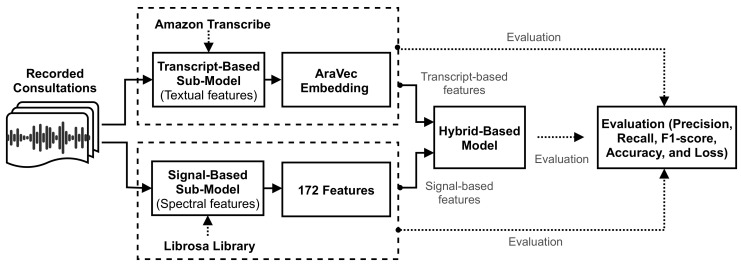
A schematic overview of the conducted methodology.

This section describes the structural implementation of the methodology. Primarily, it includes a description of the three approaches used, then describes the experimental setting and the evaluation criteria. The three approaches are: signal-based, transcript-based, and hybrid-based. The first studies the signals at the level of statistical characterization. The second converts the audio recordings into text then deploys a deep learning model to extract features from the text. The third approach combines the statistical and text-based features. Figure 4 presents an overview of the methodology, while the following subsections describe the three approaches in more detail.

### 5.1. Data Description

The raw dataset consists of 2,138 labeled recordings of consultations. The consultations consist of two speakers, the doctor and the patient. The dataset was labeled manually by the operations team based on predefined quality indicators. The class label is a categorical attribute that has ordered values from 1 to 5 that indicate the quality, where 5 refers to the highest quality. The dataset is multi-class and highly imbalanced, hence, another version of the dataset was constructed where the class label is binary. Thereby, the fourth and fifth classes were combined into a single class representing the negative class, comprising the recordings of high quality. The first, second, and third classes were combined into the positive class, which refers to the recordings of low quality.

The retrieved recordings from the database needed to be processed and formulated in a shape that can be accepted by the machine/deep learning algorithms. Therefore, two different feature sets were extracted from the recordings: which are spectral and text-based features. To illustrate, the recordings were fed into different algorithms to extract various statistical and spectral attributes from the acoustic signals of the recordings. Additionally, the recordings were transcribed, and text-based features were extracted from the transcripts. The spectral features of the signals include the Mel spectrogram, MFCCs, ZCR, and other meta-features which indicate the ratio of symptom words, the ratio of stopwords, and the number of tokens. On the other hand, the text-based features were extracted based on the word embedding model using the “AraVec” model. Hence, each recording was substituted by 172 spectral features and features from the embeddings (of a length depending on the embedding dimension), while the last column of the dataset represents the class label.

### 5.2. Signal-Based Approach

The processed signals were audio recordings of medical consultations carried out by Altibbi’s doctors and patients. The duration of a recording was maximally 20 min, where they were sampled at 8,000 Hz, and they were monophonic (one channel). Extracting spectral features from the speech signals demands that they be prepared in an appropriate form. Hence, the recordings were divided into segments of a length of 60 s. Three distinct types of features were extracted from each segment, then averaged over; in other words, audio of 15 min was divided into 15 segments, each of 1 min duration. The number of samples in each segment is given by Equations (5)–(7). In Equation (5), the total number of samples in a recording is defined by the duration of the recording multiplied by the sampling rate. Equation (6) computes the number of segments needed to divide the audio, which is measured in seconds. Equation (7) calculates the number of samples per segment.

(5) No.ofsamples=sampling_rate×duration

(6) $$No.\;of\;segments=\frac{duration\;(s)}{segment\;length\;(s)}$$

(7) $$Samples\;per\;segment=\frac{samples\;per\;audio}{No.\;of\;segments}$$

Accordingly, all segments have the same number of samples except the last one that has all the remaining samples of the division operation. The features extracted from the 15 segments were averaged and the resulting average vector represented the corresponding audio file.

#### 5.2.1. Feature Extraction

Essentially, three different types of features were used, and are widely used in the literature with audio analysis: the MFCCs, the spectrogram, and the zero-crossing rate. Three other manually computed features were also used, which are the number of tokens in the audio transcript, the ratio of symptom words in the transcript, and the ratio of stopwords. The MFCCs features were subject to 40 features resulting from 40-band filter banks. Those features were the MFCCs, the delta, and delta-delta features that reveal the rate and acceleration of the speech. The spectrogram represents the variations of the frequencies in terms of magnitude or power over time. It is calculated using filter banks that simulate the perception scale of the cochlea of the human ear (“Mel scale”) by a mapping from the linear frequency domain to the perceptual (non-linear) domain (see Equation (8)).

(8) $$Mel\left(f\right)=1,127\times log_{2}\left(1+\frac{f}{700}\right)$$

These filters are short-time Fourier transform (STFT) filters that dissect the signals into components of frequency sub-bands at a certain time. The window size of the STFT filters was 128 samples, resulting in a spectrogram of 128 pins of frequency, where the number of filters was 2048, the hop-length was 512, and the window type was Hann. The Mel spectrogram acted as a feature extractor, where the resulting vector of 128 bins represented the spectral information that could be modeled in the same way by the human ear and match the resolution of the human auditory system.

The zero-crossing rate feature encompasses the rate of change of the signal between positive and negative values, which means how many times the signal crosses the *x*-axis. This was utilized to manifest the silent periods during the consultation, given that unvoiced speech signals have a high value of the zero-crossing rate. It was calculated with a frame-length of 2048 and a hop length of 512. Nonetheless, three additional features were also extracted, which are meta-features depending on the transcripts of the consultations. The first shows the number of unique tokens in the respective transcript to represent the length of the consultation. The second presents the ratio of symptom words in the transcript. The symptom terms were retrieved from the database and searched for in transcripts, and calculated by Equation (9), where the number of symptoms is the matched symptoms with the list of symptoms retrieved from the database in the corresponding audio transcript, and the unique tokens of the corresponding transcript, as well. The ratio of symptom words can indicate the quality, where the doctor identifies the symptoms for the diagnosis.

(9) $$Symptom_{ratio}=\frac{No.\;of\;symptoms}{No.\;of\;unique\;tokens}$$

**Figure 5:**
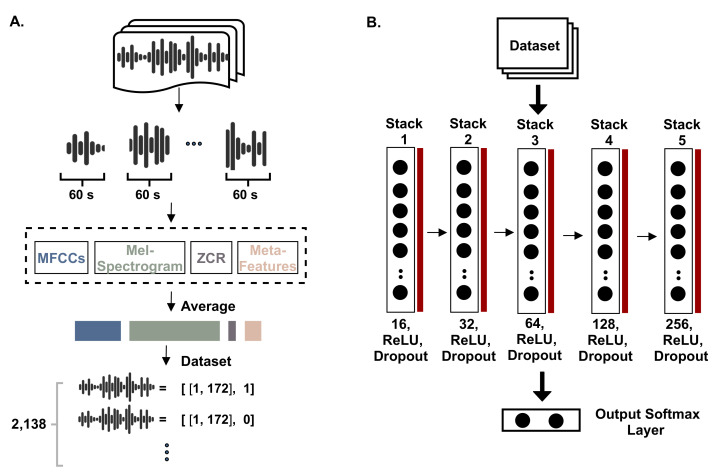
An illustration of implementing the first approach. In (**A**), the dataset is preprocessed and created, while (**B**) shows the utilized DNN model.

Finally, the third feature was the ratio of stopwords in a transcript. It was calculated by dividing the number of stopwords over the number of unique tokens in the respective transcript. This is intended to reveal how much the conversation diverts from giving useful information. Figure 5A shows the process of constructing the dataset based on the MFCCs, Mel spectrogram, zero-crossing rate (ZCR), and the meta-features of the number of tokens, the ratio of symptom words, and the stopwords ratio. Figure 5B shows the constructed deep learning model to process the signals (this is discussed further in the following subsection).

**Figure 6:**
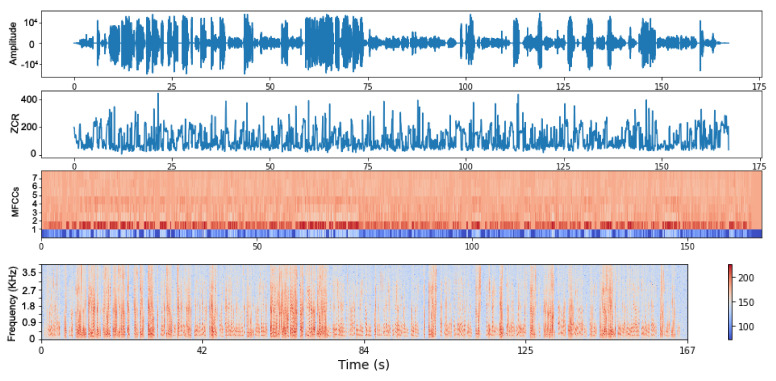
A representation of a consultation’s acoustic signal. The first plot is the signal in time domain, the second is the ZCR, the third shows the MFCCs (showing the first 8 coefficients), and the fourth is the spectrogram representation.

Figure 6 depicts the signal of a consultation in the time domain in terms of the amplitude over the time represented in seconds, then the ZCR representation of it, the MFCCs vectors, and finally the spectral envelope, which shows the frequency over time.

#### 5.2.2. Model Structure

The resulting dataset for signal-based analysis contains 172 features of the MFCCs, the Mel spectrogram, the zero-crossing rate, and the other three meta-features. Meanwhile, the number of consultations that were labeled and retrieved is 2,138. The classes indicate the quality of the consultations in a range from 1 to 5. The prepared dataset was divided into three sets: the training, the testing, and the validation dataset. The training and validation data were used to train and tune a stack of the deep feed-forward neural network with a different number of neurons at each layer, as shown in Figure 5B. The stacked layers were of different sizes of 16, 32, 64, 128, and 256, which were also dropped out by a ratio of 25% to avoid overfitting. Each layer was activated by the ReLU function and regularized by a L1-regularizer that regulated the network’s weights to avoid overfitting. The last layer of the network was a Softmax layer that generated the probabilities of each class. The Softmax function is given by Equation (10), in which *z* is the weighted sum of the input at layer *l*, *j* represents the number of neurons at the current layer, and *k* indicates the number of neurons in the previous layer.

(10) $$a_{j}^{l}=\frac{e^{z_{j}^{l}}}{\sum_{k}e^{z_{k}^{l}}}$$

### 5.3. Transcript-Based Approach

This approach studies the quality of the consultations by analyzing their transcripts. The transcripts of the consultations were extracted using Amazon, where they were then processed and text-based features wree extracted. The constructed dataset based on the transcripts was then used to train a hybrid convolution neural network and feed-forward neural network model. This is illustrated in detail in the following subsections.

#### 5.3.1. Feature Extraction

**Figure 7:**
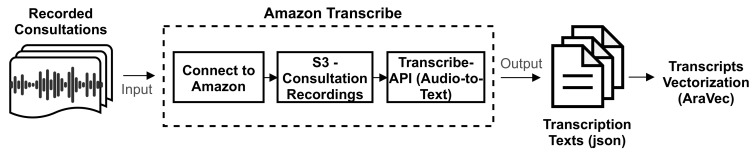
The process of converting the acoustic recordings into text and then extracting text-based features using AraVec.

Firstly, extracting the transcripts of consultation recordings was done using the Amazon transcription service (“Amazon Transcribe”). Figure 7 presents the steps of calling Amazon APIs. Particularly, this requires storing the consultations in Amazon’s Simple Storage Service (S3), then connecting to Amazon via secret and access tokens. The results of calling the APIs are text presented in “JSON” format, while the resulting transcripts are prepared for feature extraction. The extraction of text-based features from transcripts was performed by a pre-trained word embedding model called “AraVec” [39]. AraVec is an open-source pre-trained set of models for word embedding for Arabic NLP. AraVec encompasses twelve different models based on the Arabic language and data collected from Twitter and Wikipedia. The total number of used vocabularies for the development of AraVec was 3,300,000,000, where all were utilized to create two types of word embedding: the skip-gram and continuous bag-of-words (CBOW) models. The word embedding in the deep learning model was implemented by an embedding layer. The embedding layer acted as a lookup table with input and dimension lengths. The input length was the size of the unique vocabulary of a transcript (the length of a sequence). Whereas, the dimension length was a hyperparameter specifying the length of the embedding. Indeed, each word in the transcript had an embedding representation characterizing its context and semantics. In this regard, the pre-trained model used in this approach was the one trained on Twitter with either skip-gram or CBOW structures.

#### 5.3.2. Model Structure

**Figure 8:**
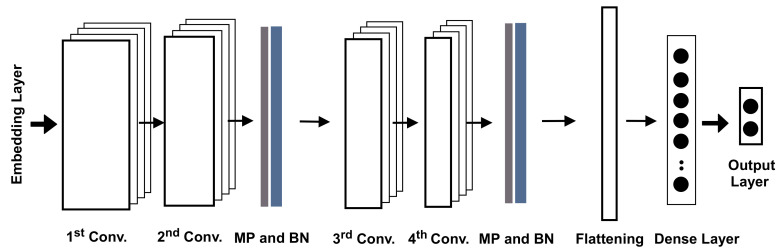
The structural design of the second approach. MP is the max-pooling operation, and BN is the batch normalization layer.

The new dataset comprises 2,138 labeled consultations with text-based features extracted from the transcripts based on AraVec. The training and validation parts of the dataset were used to train a hybrid model comprising convolutional neural networks and deep feedforward neural networks, as illustrated in Figure 8. The model had four stacked convolution layers, then a fully connected dense layer. The first two convolution layers had 32 filters with size 5 followed by max-pooling, dropout, and batch normalization layers, while the second two layers had 64 filters of size 5 and max-pooling, dropout, and batch normalization layers, as well. All were activated by the ReLU function, while the batch normalization was intended to normalize the input at each layer and regulate the gradients through layers. The output of the convolution was flattened to match the input format of the following dense fully connected layer with 400 neurons. The last layer was the output layer, which had two neurons for generating the predictions of the classes based on the Softmax function.

### 5.4. Hybrid Approach Combining Spectral Features and Transcript Features

**Figure 9:**
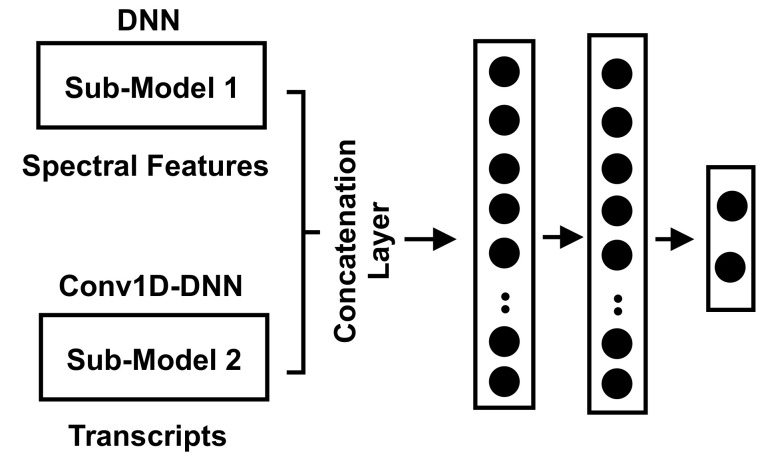
The structure of combining the two approaches of the signal-based submodel and the transcript-based submodel.

The hybrid approach combines the statistical (spectral) features of acoustic signal-based consultations and the text-based features extracted from the transcripts. This hybrid dataset was split into training, validation, and testing, where the validation data were used to optimize the created model, and the testing dataset was used to evaluate the model. Figure 9 shows the structure of the developed model, which mainly combines the signal-based and the transcript-based models and their outputs, where the first submodel is a stacked DNN, and the second submodel is a stacked Conv1D-DNN. The concatenation of the outputs from the two submodels was done by adopting a concatenation layer that takes a list of tensors and outputs a single tensor. The output of the concatenation layer was fed into two stacked dense layers, where both had 128 neurons. The last layer was the output layer to produce the probabilities of the output classes.

### 5.5. Experimental Settings

Regarding the system and hardware settings, the operating system used was Ubuntu (18.04), the development platform was PyCharm IDE, the processor was an Intel(R) Core(TM) i7-1065G7 CPU, and the random access memory (RAM) was 20 GB. The Python language environment was version 3.7 and the deep learning framework used was TensorFlow [40] based on Keras APIs. All consultation recordings (the digital signals) were processed, analyzed, and features extracted using the Librosa library [41].

For the embedding model, the utilized models were deployed at dimension 300 of AraVec pre-trained models using the Gensim library [42]. Preparation of the textual transcripts consisted of various processes, including padding texts using the “Post” parameter to the maximum length of all transcripts. Further, all values were normalized using the min–max approach. Meanwhile, the DNN and Conv1D-DNN models were developed using a sequential stack of layers. The proposed models were trained using 60% of the data and were validated and tested using two different distinct subsets, each of which represented 20% of the data. The models were trained using the “Binary_crossentropy” loss function. The weights of the network were learned using an adaptive learning optimizer, which represents the adaptive moment estimation (“Adam”) optimizer. The optimizer’s learning weight parameter was set to a fixed learning rate of 5 × 10${}^{-6}$. This learning rate was chosen based on multiple experiments at different learning rates from 10${}^{-3}$ to 10${}^{-8}$, where the best performance was obtained when the learning rate was 5 × 10${}^{-6}$. Moreover, the training was conducted using the batch strategy, which was adopted with a size of 128. Additionally, as the data was imbalanced and the minor class was the class of interest, it was handled utilizing a cost-sensitive approach that uses weight parameters for each class, where they act as penalty parameters while training.

### 5.6. Evaluation Criteria

Different evaluation measures were utilized for the assessment of the designed model: precision, recall, F1-score, accuracy, and loss of the optimizer. The metrics were calculated and presented in terms of the macro-average, and for the positive class. The accuracy was considered to be the ratio of correct predictions over the total number of consultations ($n_{s}$), as defined in Equation (11), where *y* is the actual value of consultations (*i*), and $\hat{y}$ is the predicted value.

(11) $$Accuracy\left(y,\hat{y}\right)=\frac{1}{n_{s}}\sum_{i=0}^{n_{s}-1}1\left({\hat{y}}_{i}=y_{i}\right)$$

The macro-recall computes the average recall of each class, for which the recall implies how much the model can identify the positively classified consultations. The macro-recall is defined by Equation (12), in which *L* is the set of all classes, $y_{l}$ is the proportion of predicted consultations with label *l*, and ${\hat{y}}_{l}$ represents the consultations that have true labels.

(12) $$Recall=\frac{1}{\left|L\right|}\sum_{l\in L}R\left(y_{l},{\hat{y}}_{l}\right),\;\;\;R\left(y_{l},{\hat{y}}_{l}\right)=\frac{\left|y_{l}\cap {\hat{y}}_{l}\right|}{\left|{\hat{y}}_{l}\right|}$$

Similarly, the macro-precision calculates the mean precision across all classes. In this case, the precision demonstrates the ratio of correctly identified positive consultations over the actual number of positive consultations (Equation (13)).

(13) $$Precision=\frac{1}{\left|L\right|}\sum_{l\in L}P\left(y_{l},{\hat{y}}_{l}\right),\;\;\;P\left(y_{l},{\hat{y}}_{l}\right)=\frac{\left|y_{l}\cap {\hat{y}}_{l}\right|}{\left|y_{l}\right|}$$

The macro F1-score (F1-score) computes the score for each class and then returns their unweighted average. The F1-score is also known as the harmonic mean of precision and recall and shows the balance between them. The mathematical formula of the F1-score is defined by Equations (14) and (15), where *P* is the precision, *R* is the recall, and β is a weighting parameter.

(14) $$F1-score=\frac{1}{\left|L\right|}\sum_{l\in L}F_{\beta }\left(y_{l},{\hat{y}}_{l}\right)$$

(15) $$F_{\beta }\left(y_{l},{\hat{y}}_{l}\right)=\left(1+\beta^{2}\right)\frac{P\left(y_{l},{\hat{y}}_{l}\right)\times R\left(y_{l},{\hat{y}}_{l}\right)}{\beta^{2}P\left(y_{l},{\hat{y}}_{l}\right)+R\left(y_{l},{\hat{y}}_{l}\right)}$$

## 6. Results

This section describes the obtained results of the signal-based approach, the transcript-based approach, and the hybrid of both. The best results are highlighted in bold typeface.

### 6.1. Signal-Based Results

This subsection analyzes the results of the prediction of the quality of consultations based solely on the spectral features. Particularly, the results are discussed over two stages—first considering the MFCCs alone, and second combining a set of statistical and spectral features. Table 1 displays the results of quality prediction depending on features obtained from MFCCs only, where the table shows the precision, recall, and F1-score in terms of the macro-average, and for the positive class (the consultations of low quality), as well as the accuracy and loss. The proposed stacked deep learning model was evaluated at six different learning rates (i.e., $1\times 10^{-3}$, $5\times 10^{-3}$, $1\times 10^{-4}$, $5\times 10^{-4}$, $1\times 10^{-6}$, and $5\times 10^{-6}$). Regarding the precision, it is clear that the best precision for the positive class and the macro-average was obtained when the learning rate was $5\times 10^{-4}$, yielding 52% and 58.8%, respectively. Meanwhile, there was no obvious trend for the precision with the learning rate, where decreasing it deteriorated the performance, and increasing it did not result in better results. The best recall of the positive class was obtained by a value of 100% at a learning rate of $1\times 10^{-6}$. The best recall in terms of macro-average was 57.7% and was achieved at a learning rate of $5\times 10^{-4}$. The same scenario was seen for the F1-score, where the highest for the positive class was at $1\times 10^{-6}$ (57%), and for the macro-average it was 57.7% at $5\times 10^{-4}$. Regarding the accuracy, the best value (61.4%) was obtained when the learning rate was $5\times 10^{-4}$. The minimal loss was 0.688 when the learning rate was $5\times 10^{-3}$. It can be concluded that when the learning rate was $5\times 10^{-4}$, the best results in terms of precision, recall, F1-score, and accuracy were obtained. Thus, further analysis was conducted in this setting.

Table 2 presents the performance results of quality prediction when considering the total number of statistical and spectral features. It is clear from the table that the best-obtained precision regarding the positive class and the macro-average was when the learning rate was $5\times 10^{-4}$, which were 48.5%, and 58.9%, respectively. Meanwhile, at a learning rate of $5\times 10^{-6}$, the same macro-precision was obtained (58.9%), while for the positive class it was slightly less (44.8%). In terms of the recall, the positive class had 100% percent recall at $1\times 10^{-3}$ and $1\times 10^{-6}$. Meanwhile, the positive class had 59.2% macro-recall at $5\times 10^{-4}$. Regarding the F1-score, the highest of the macro-average was 58.2% at $5\times 10^{-4}$, and for the positive class it was 57.8% at $5\times 10^{-6}$. For the accuracy, it was best with a ratio of 60.1% at a learning rate of $1\times 10^{-4}$. For the loss, it was best obtained at $5\times 10^{-4}$ with a minimum of 0.691.

**Figure 10:**
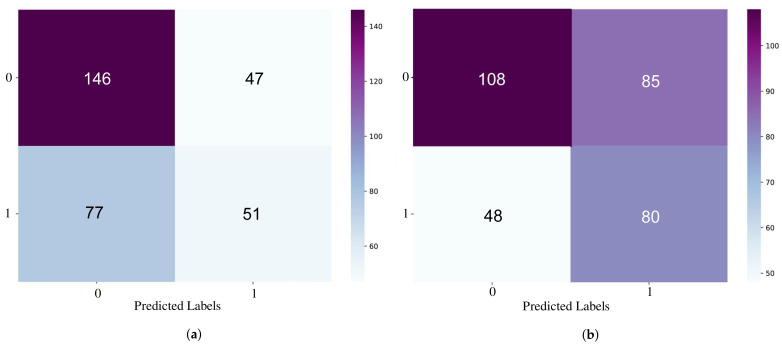
The heatmap representation of the confusion matrix of the best models obtained from using the MFCCs alone (**a**) and using the combination of all spectral features (**b**).

When comparing the results of the MFCC features alone or the complete set of features together, it can be seen that they differed slightly. For example, the best precision of the positive class was obtained when the model relied only on the MFCCs (52%), however, it was 48.5% for the complete set of features. However, if considering the macro-average of the F1-score, it was better in the case of the total set of features (58.2%), and for the MFCCs it was 57.7%. Figure 10 shows the confusion matrices represented as heatmaps of the best-obtained models when considering the MFCCs (Figure 10a), or the total number of spectral features (Figure 10b). The *x*-axis is the predicted labels, and the *y*-axis is the true labels.

### 6.2. Transcript-Based Results

This subsection discusses the results obtained from the features extracted based on the created transcripts from Amazon’s Transcribe. Table 3 shows the performance results of the proposed “Conv1D-DNN” based on four different embedding models (Twitter or Wiki at CBOW or SG) at different embedding dimensions (100 or 300) and different learning rates ($1\times 10^{-4}$, $5\times 10^{-4}$, $5\times 10^{-6}$). Regarding the precision, the best-attained precision of the positive class was 44% from AraVec-Twitter-CBOW when the embedding dimension was 100 and the learning rate was $5\times 10^{-6}$. In this setting, in contrast, the recall and F1-score were 9.6% and 15.8%, respectively. For the macro-averaged precision, the model performed best (67.8%) when the embedding model was AraVec-Twitter-SG and the dimension was 300 at a learning rate of $5\times 10^{-4}$. Markedly, other embeddings could achieve reasonably good results. For instance, Twitter-CBOW (dimension=100) had 54.6%, Twitter-CBOW (dimension = 300) achieved 50.1%, and WiKi-CBOW (dimension = 100) achieved 51.8%.

Regarding the recall, for the positive class different models achieved 100% (e.g., Twitter with CBOW or SG structures) at both dimensions, and the WiKi-SG achieved this at dimension 100. Further, the Twitter-CBOW (dimension = 300) achieved a recall of 54.4%. In terms of the macro-average of recall, even though all the models achieved relatively similar results with slight differences, WiKi-CBOW at dimension 100 achieved the best with a percentage of 51.9%. Regarding the F1-score, Twitter-SG outperformed the others, even though Twitter-CBOW achieved a very similar result. Twitter-SG attained an F1-score of 52.5% for the positive class at dimension 300 and learning rate of $5\times 10^{-4}$. Meanwhile, WiKi-CBOW at dimension 100 was best in terms of macro-average (51.8%). Considering the accuracy, Twitter-CBOW (300), Twitter-SG (100 and 300), and WiKi-(SG and CBOW) at dimension 100 achieved the best results of 64.5%. However, Twitter-CBOW (100) and WiKi-SG (100) accomplished slightly less, reaching 63.6%. The minimal loss values were achieved with Twitter-CBOW of dimension 300, with a value of 3.297. Roughly speaking, all models achieved relatively similar results, but if we consider the precision of the positive class then Twitter-CBOW (100) was the best, while if we consider the F1-score of the positive class then it was Twitter-SG with dimension 300. Therefore, the two models were evaluated further at different vocabulary sizes, and the results are presented in Table 4.

Table 4 shows the results of considering the features extracted from the transcripts with two different embedding models: Twitter (SG and CBOW) and WiKi-CBOW, which were superior to the other models, as shown in the previous table. In this table, three vocabulary sizes were evaluated: 9000, 18,000, and 27,000 of the most frequent words. Regarding the precision of the positive class, the best results were obtained with AraVec-Wiki-CBOW at dimension 100, with a value of 36.5%. For the macro-average, the best value was obtained by AraVec-Twitter-SG (300) with a value of 62.6%. It can be seen that AraVec-WiKi-CBOW could also achieve a relatively very good macro-average of precision (55.8%). Considering the recall, the best-obtained result for the positive class was 100%, obtained by AraVec-Twitter (CBOW or SG) at dimension 100, when the vocabulary size was 9,000. However, WiKi-CBOW and Twitter-SG accomplished high results for the positive class (99.1%). Regarding the macro-average of recall, the best results were obtained for the WiKi-CBOW when the vocabulary size was 27,000, with a value of 52%. Other models at various vocabulary sizes achieved similar results, even though there was no clear increasing or decreasing trend of recall when the vocabulary size of the embedding layer was changed. Investigation of the performance in terms of the F1-score shows that Twitter-SG performed the best, with a score of 53.1% for the positive class at a vocabulary size of 9,000. However, the other models at vocabulary size 9,000 accomplished similar results, about 52%. Considering the macro-average of the F1-score, the best results were obtained by Twitter-CBOW (100), with a value of 44.3%. In terms of accuracy, Twitter-CBOW and Twitter-SG (100 and 300) achieved the best result of 64.5%. Regarding the loss, the best was achieved by Twitter-SG (300), with a value of 4.556. Roughly, considering the results of the F1-score, we can say that Twitter (CBOW or SG) at dimensions 100 and 300, respectively, performed the best.

**Figure 11:**
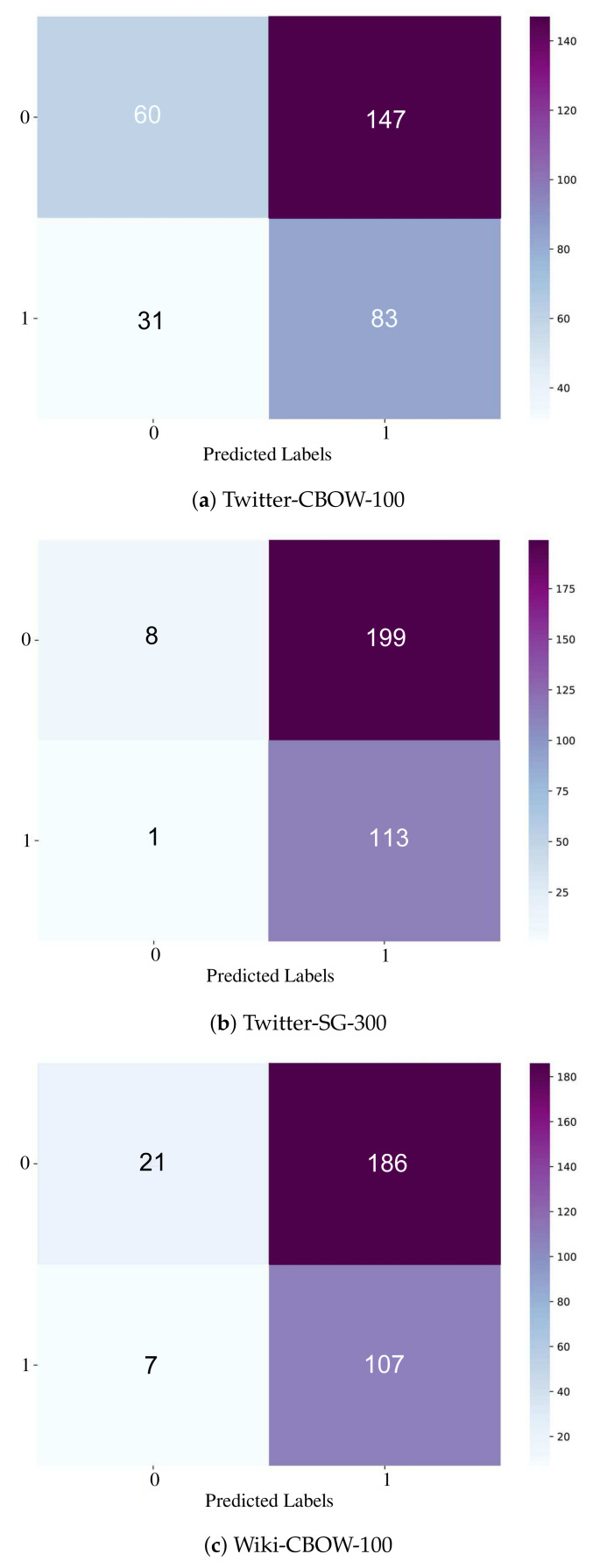
A heatmap representation of the confusion matrices of the best models from the transcript-based approach with different embedding models.

Figure 11 shows heatmap representations of the best-performing models based on the transcript-only approach. Figure 11a shows Twitter-CBOW (dimension = 100). Figure 11b shows Twitter-SG at dimension 300, while Figure 11c presents WiKi-CBOW at dimension 100. It can be seen that Twitter-CBOW (100) gave the maximum balance of the true positive, true negative, false positive, and false negative. Hence, this model was selected for any additional experiments.

### 6.3. Hybrid-Based Results

This subsection discusses the results of combining the features from the transcripts and from the signals analysis (spectral features). Twitter-CBOW (100) and Twitter-SG (300) were used to process the data from the transcripts, while all statistical and spectral features were used from the signal-based part. Table 5 shows the results of the hybrid model at a vocabulary size of 9,000, or when considering all the vocabularies, at different learning rates ($5\times 10^{-4}$, $9\times 10^{-4}$, and $5\times 10^{-5}$) and different batch sizes (125, 64, and 32). The last three experiments were considered with all the vocabularies, and at three different epochs (30, 50, and 100). When the embedding weights were trainable, the embedding model was Twitter-CBOW, the embedding dimension was 100, the learning rate was $5\times 10^{-5}$, and the batch size was 128. Regarding the precision, the best obtained precision of the positive class was 41.1% for Twitter-CBOW (dimension = 100) with a learning rate of $5\times 10^{-5}$ and batch size of 64. For the macro-average, it was best (51.3%) for Twitter-CBOW (100) when the learning rate was $9\times 10^{-4}$ with a batch size of 32. In terms of the recall, the Twitter-SG achieved better results (100%) when the vocabulary size was 9,000 at either $9\times 10^{-4}$ or $5\times 10^{-5}$ learning rates, and with all vocabularies when the learning rate was $5\times 10^{-4}$. The best recall in terms of the macro-average was 51.3% and accomplished by Twitter-CBOW (100) at a learning rate of $5\times 10^{-5}$ with a batch size of 64. For the F1-score of the positive class, the best was obtained in similar conditions to the recall, but the best value was 57%. Additionally, the best F1-score of the macro-average was 50.7% with Twitter-CBOW (100) and the learning rate was $5\times 10^{-5}$ with a batch size of 64. Moreover, regarding the accuracy, the best-obtained percentage was 60.1% for Twitter-CBOW (100) with a learning rate of $9\times 10^{-4}$ and a batch size of 128. The minimal loss was 0.932, obtained by Twitter-CBOW (100) with a learning rate of $5\times 10^{-5}$ and a batch size of 128. It can be concluded from the table that increasing the number of epochs with trainable embedding weights did not improve the performance of the model.

**Figure 12:**
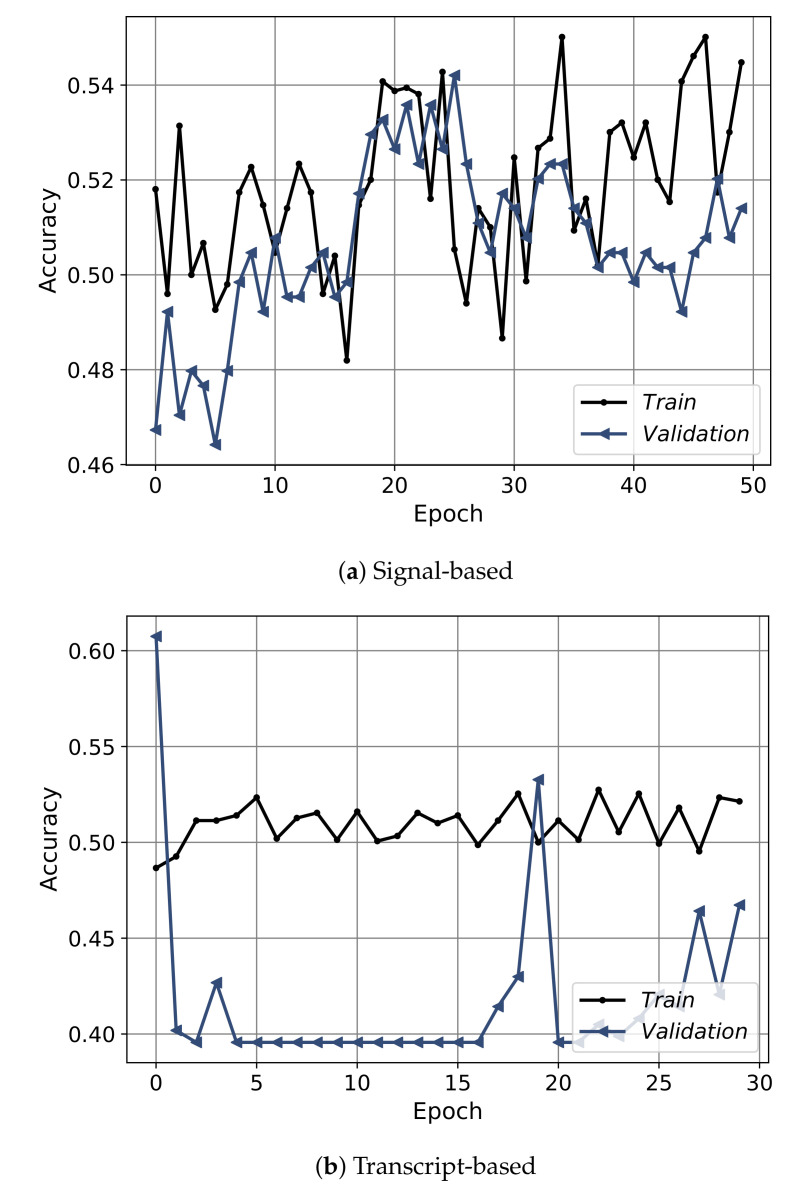

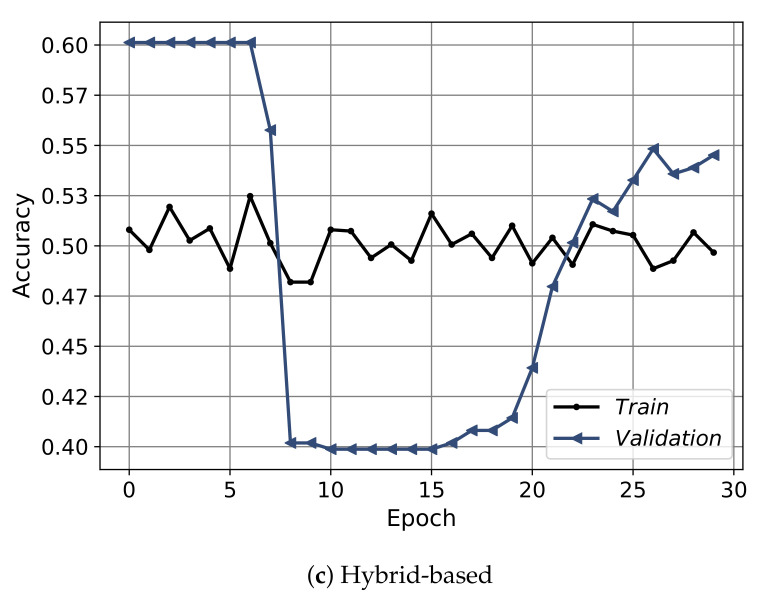
The convergence curves in terms of the accuracy of the best models for the three approaches using the spectral features only, the transcript features only, and the hybrid of both.

**Figure 13:**
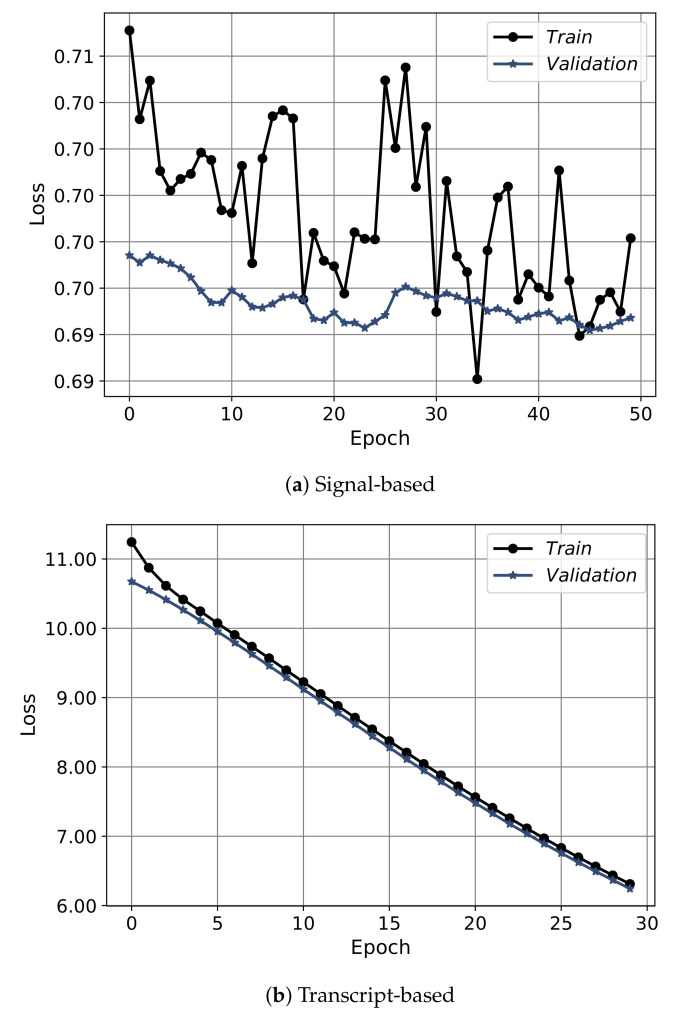

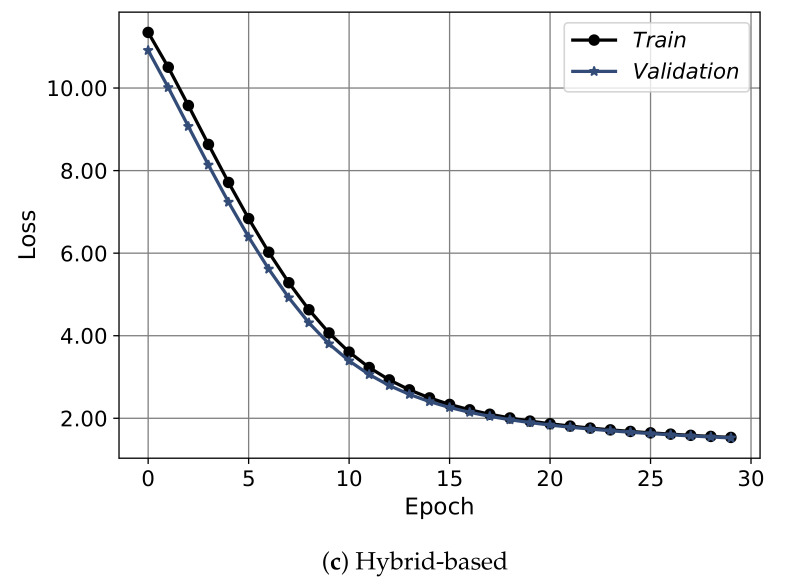
The convergence curves in terms of the loss of the best models for the three approaches of using the spectral features only, the transcript features only, and the hybrid of both.

Figure 12 shows the convergence curves of the best models obtained from each approach: the signal-based, the transcript-based, and the hybrid model. The convergence shows the epochs on the *x*-axis and the accuracy on the *y*-axis. The convergence is depicted for training (black) and validation (blue), the signal-based is shown for 50 epochs, and the transcript-based/hybrid models plotted over 30 epochs. From the figure it is clear that the convergence of the signal-based approach had an obvious increasing trend up to thirty epochs then the model started to slightly overfit. For the transcript-based and hybrid approaches, the models did not converge smoothly. Furthermore, Figure 13 shows the convergence in terms of the loss. Regarding the signal-based approach, the model showed a highly fluctuating convergence trend over the course of epochs, even though it was exhibiting a decreasing behavior. For the transcript-based and hybrid approaches the curves were smoother than for the signal-based approach, even though the model in the transcript-based approach was steeper. Moreover, the training and validation of the loss-convergence curves of the transcript-based and hybrid approaches were very close to each other, which implies less overfitting.

From the three approaches, it can be seen that when relying on the signal-based analysis only, the model obtained the highest precision of 52%. Meanwhile, combining the features from the signals and the transcripts as in the hybrid approach did not result in a better precision. Depending only on spectral and statistical features is not able to reliably judge the quality of the consultations since the speech characteristics differ among people and depend on many factors, such as gender. However, the transcript-based and the hybrid models could still obtain good results, even though this behavior is expected since the transcription process is not optimal and still needs improvement, which in turn would further improve the precision of the transcript-based and hybrid models. Additionally, increasing the dataset size might improve the performance.

## 7. Conclusions

This paper presented a deep learning approach for assessing the quality of medical consultations based on recordings stored and labeled by the Altibbi company. The automatic assessment of consultations is important in order to maintain a high quality of service and to decrease the time needed to listen to the recorded consultations and grade them. This paper proposed an automatic quality assessment model for Altibbi’s auditory consultations based on three approaches: a signal-based model, a transcript-based model, and a hybrid one. The signal-based approach extracted different spectral and statistical features and fed them into the stacked layers of a neural network. For the transcript-based approach, the text-based features were extracted from transcripts using pre-trained embedding models and fed into the stacked layers of convolution and deep neural networks. We also evaluated a model which was the hybrid of the two (signal-based and transcript-based). All conducted experiments were evaluated in terms of the precision, recall, F1-score, accuracy, and loss for the positive class, and the macro-average of the two classes. The signal-based approach achieved the highest precision compared to the other approaches, with a value of 52%. Overall, the proposed models accomplished encouraging, good results, and they improved the precision of the traditional approach followed by Altibbi’s operations team. The automatic quality assessment of medical consultations is important, especially for a complex language such as Arabic. However, it is necessary to build on this research to improve it, where further areas of development could include improving the transcription process, increasing the size of the labeled dataset, and implementing advanced language models to handle the transcripts. Additional spectral features might also be investigated and explored.

## Figures and Tables

**Table 1:** Comparison of performance measures based on the MFCC features alone (P.C. is the positive class, L.R. is the learning rate, and Mc. Avg. is the macro-average).

Precision		Recall		F1-score		Accuracy	Loss	L.R.	Model
P.C.	Mc. Avg.	P.C.	Mc. Avg.	P.C.	Mc. Avg.				
0.000	0.301	0.000	0.500	0.000	0.375	0.601	6.116	1 × 10${}^{-3}$	Stacked DNN
0.463	0.551	0.438	0.550	0.449	0.551	0.573	**0.688**	5 × 10${}^{-3}$	
0.000	0.301	0.000	0.500	0.000	0.375	0.601	6.116	1 × 10${}^{-4}$	
**0.520**	**0.588**	0.398	**0.577**	0.451	**0.577**	**0.614**	0.690	5 × 10${}^{-4}$	
0.399	0.199	**1.000**	0.500	**0.570**	0.285	0.399	9.221	1 × 10${}^{-6}$	
0.401	0.502	0.367	0.502	0.384	0.502	0.530	0.692	5 × 10${}^{-6}$	

**Table 2:** Comparison of performance measures based on the combination of MFCCs, Mel spectrogram, ZCR, and meta-features (P.C. is the positive class, L.R. is the learning rate, and Mc. Avg. is the macro-average).

Precision		Recall		F1-score		Accuracy	Loss	L.R.	Model
P.C.	Mc. Avg.	P.C.	Mc. Avg.	P.C.	Mc. Avg.				
0.398	0.199	**1.000**	0.500	0.570	0.285	0.399	9.221	1 × 10${}^{-3}$	Stacked DNN
0.455	0.557	0.586	0.560	0.512	0.551	0.555	0.695	5 × 10${}^{-3}$	
0.000	0.301	0.000	0.500	0.000	0.375	**0.601**	6.116	1 × 10${}^{-4}$	
**0.485**	**0.589**	0.625	**0.592**	0.546	**0.582**	0.586	**0.691**	5 × 10${}^{-4}$	
0.399	0.199	**1.000**	0.500	0.570	0.285	0.399	9.221	1 × 10${}^{-6}$	
0.448	**0.589**	0.813	0.575	**0.578**	0.519	0.526	0.694	5 × 10${}^{-6}$	

**Table 3:** The results of adopting the transcripts only by using the Conv1D-DNN model at different embedding models and dimensions, and at different learning rates, regarding the precision, recall, and F1-score of the positive class and the macro-average of the classes (E.D. is the embedding dimension).

Embedding Model	Precision		Recall		F1-score		Accuracy	Loss	E.D.	L.R.
	P.C.	Mc. Avg.	P.C.	Mc. Avg.	P.C.	Mc. Avg.				
AraVec-Twitter-CBOW	0.355	0.178	**1.000**	0.500	0.524	0.262	0.355	71.637	100	1 × 10${}^{-4}$
AraVec-Twitter-CBOW	0.355	0.178	**1.000**	0.500	0.524	0.262	0.355	6.494		5 × 10${}^{-4}$
AraVec-Twitter-CBOW	**0.440**	0.546	0.096	0.514	0.158	0.463	0.636	4.999		5 × 10${}^{-6}$
AraVec-Twitter-CBOW	0.355	0.178	**1.000**	0.500	0.524	0.262	0.355	82.690	300	1 × 10${}^{-4}$
AraVec-Twitter-CBOW	0.000	0.322	0.000	0.500	0.000	0.392	**0.645**	4.419		5 × 10${}^{-4}$
AraVec-Twitter-CBOW	0.356	0.501	0.544	0.501	0.431	0.484	0.489	**3.297**		5 × 10${}^{-6}$
AraVec-Twitter-SG	0.355	0.178	**1.000**	0.500	0.524	0.262	0.355	78.438	100	1 × 10${}^{-4}$
AraVec-Twitter-SG	0.355	0.178	**1.000**	0.500	0.524	0.262	0.355	6.406		5 × 10${}^{-4}$
AraVec-Twitter-SG	0.000	0.322	0.000	0.500	0.000	0.392	**0.645**	5.240		5 × 10${}^{-6}$
AraVec-Twitter-SG	0.000	0.322	0.000	0.500	0.000	0.392	**0.645**	84.065	300	1 × 10${}^{-4}$
AraVec-Twitter-SG	0.356	**0.678**	**1.000**	0.502	**0.525**	0.267	0.358	4.355		5 × 10${}^{-4}$
AraVec-Twitter-SG	0.295	0.465	0.114	0.482	0.165	0.446	0.589	3.526		5 × 10${}^{-6}$
AraVec-WiKi-SG	0.355	0.178	**1.000**	0.500	0.524	0.262	0.355	1.420	100	1 × 10${}^{-4}$
AraVec-WiKi-SG	0.000	0.321	0.000	0.495	0.000	0.390	0.639	5.856		5 × 10${}^{-4}$
AraVec-WiKi-SG	0.000	0.322	0.000	0.500	0.000	0.392	**0.645**	4.662		5 × 10${}^{-6}$
AraVec-WiKi-CBOW	0.000	0.322	0.000	0.500	0.000	0.392	**0.645**	79.096	100	1 × 10${}^{-4}$
AraVec-WiKi-CBOW	0.000	0.322	0.000	0.500	0.000	0.392	**0.645**	6.471		5 × 10${}^{-4}$
AraVec-WiKi-CBOW	0.378	0.518	0.395	**0.519**	0.386	**0.518**	0.555	5.008		5 × 10${}^{-6}$

**Table 4:** The results of adopting the transcripts only by using the Conv1D-DNN model at a learning rate of 5 × 10${}^{-4}$, and at varying vocabulary sizes (E.D. is the embedding dimension).

Vocabs Size	Embedding Model	Precision		Recall		F1-score		Acc.	Loss	E.D.
		P.C.	Mc. Avg.	P.C.	Mc. Avg.	P.C.	Mc. Avg.			
9000	AraVec-Wiki-CBOW	0.355	0.511	0.991	0.500	0.523	0.271	0.358	5.834	100
18,000		0.261	0.449	0.053	0.485	0.088	0.420	0.611	6.895	
27,000		**0.365**	0.558	0.939	**0.520**	0.526	0.352	0.399	5.139	
9000	AraVec-Twitter-CBOW	0.355	0.178	**1.000**	0.500	0.524	0.262	0.355	6.211	100
18,000		0.361	0.510	0.728	0.509	0.483	**0.443**	0.445	6.244	
27,000		0.000	0.322	0.000	0.500	0.000	0.392	**0.645**	6.414	
9000	AraVec-Twitter-SG	0.362	**0.626**	0.991	0.515	**0.531**	0.302	0.377	**4.556**	300
18,000		0.276	0.456	0.070	0.484	0.112	0.429	0.604	5.237	
27,000		0.356	0.504	0.868	0.502	0.505	0.365	0.396	4.713	
9000	AraVec-Twitter-SG	0.355	0.178	**1.000**	0.500	0.524	0.262	0.355	6.036	100
18,000		0.000	0.322	0.000	0.500	0.000	0.392	**0.645**	6.096	
27,000		0.000	0.322	0.000	0.500	0.000	0.392	**0.645**	5.870	

**Table 5:** The results of the hybrid approach using the transcript features and spectral features using AraVec-Twitter with different embedding model structures (E.M.s), different E.D., embedding weights (E.W.s), L.R.s, and batch sizes (B.S.s).

Vocab Size	E.M.	Precision		Recall		F1-score		Acc.	Loss	Epochs	E.W.	E.D.	L.R.	B.S.
		P.C.	Mc. Avg.	P.C.	Mc. Avg.	P.C.	Mc. Avg.							
9000	SG	0.000	0.299	0.000	0.495	0.000	0.373	0.595	2.085	30	Non	300	5 × 10${}^{-4}$	128
	SG	0.399	0.199	**1.000**	0.500	**0.570**	0.285	0.399	11.683				9 × 10${}^{-4}$	
	SG	0.399	0.199	**1.000**	0.500	**0.570**	0.285	0.399	2.299				5 × 10${}^{-5}$	
All	SG	0.399	0.199	**1.000**	0.500	**0.570**	0.285	0.399	2.020	30	Non	300	5 × 10${}^{-4}$	128
	SG	0.000	0.301	0.000	0.500	0.000	0.375	**0.601**	11.685				9 × 10${}^{-4}$	
	SG	0.394	0.322	0.977	0.491	0.562	0.286	0.393	2.162				5 × 10${}^{-5}$	
All	CBOW	0.400	0.501	0.250	0.501	0.308	0.488	0.551	3.315	30	Non	100	5 × 10${}^{-4}$	128
	CBOW	0.000	0.301	0.000	0.500	0.000	0.375	**0.601**	11.616				9 × 10${}^{-4}$	
	CBOW	0.383	0.387	0.891	0.469	0.535	0.309	0.383	2.452				5 × 10${}^{-5}$	
All	CBOW	0.377	0.483	0.336	0.484	0.355	0.483	0.514	1.908	30	Non	100	5 × 10${}^{-4}$	64
	CBOW	0.408	0.510	0.586	0.511	0.481	0.495	0.495	11.623				9 × 10${}^{-4}$	
	CBOW	**0.411**	0.512	0.523	**0.513**	0.460	**0.507**	0.511	1.524				5 × 10${}^{-5}$	
All	CBOW	0.397	0.489	0.898	0.496	0.550	0.355	0.414	1.256	30	Non	100	5 × 10${}^{-4}$	32
	CBOW	0.404	**0.513**	0.820	0.509	0.541	0.42	0.445	11.636				9 × 10${}^{-4}$	
	CBOW	0.399	0.499	0.844	0.500	0.541	0.394	0.430	1.205				5 × 10${}^{-5}$	
All	CBOW	0.368	0.465	0.523	0.464	0.432	0.451	0.452	2.818	30	Trainable	100	5 × 10${}^{-5}$	128
	CBOW	0.368	0.477	0.305	0.479	0.333	0.475	0.514	**0.932**	50				
	CBOW	0.333	0.449	0.297	0.452	0.314	0.450	0.483	1.447	100				
